# An investigation into the potential association between nutrition and Alzheimer’s disease

**DOI:** 10.3389/fnut.2024.1306226

**Published:** 2024-03-07

**Authors:** Mingyue He, Tenghong Lian, Zhan Liu, Jinghui Li, Jing Qi, Jing Li, Peng Guo, Yanan Zhang, Dongmei Luo, Huiying Guan, Weijia Zhang, Zijing Zheng, Hao Yue, Wenjing Zhang, Ruidan Wang, Fan Zhang, Wei Zhang

**Affiliations:** ^1^Department of Neurology, Beijing Tiantan Hospital, Capital Medical University, Beijing, China; ^2^Center for Cognitive Neurology, Department of Neurology, Beijing Tiantan Hospital, Capital Medical University, Beijing, China; ^3^Department of Blood Transfusion, Beijing Tiantan Hospital, Capital Medical University, Beijing, China; ^4^China National Clinical Research Center for Neurological Diseases, Beijing Tiantan Hospital, Capital Medical University, Beijing, China; ^5^Center of Parkinson’s Disease Institute for Brain Disorders, Beijing, China; ^6^Beijing Key Laboratory on Parkinson Disease, Beijing, China

**Keywords:** Alzheimer’s disease, mild cognitive impairment, dementia, nutritional status, nutrition-related variables

## Abstract

**Background:**

Malnutrition is the most common nutritional issue in Alzheimer’s disease (AD) patients, but there is still a lack of a comprehensive evaluation of the nutritional status in AD patients. This study aimed to determine the potential association of various nutritional indices with AD at different stages.

**Methods:**

Subjects, including individuals with normal cognition (NC) and patients diagnosed with AD, were consecutively enrolled in this cross-sectional study. Demographics, body composition, dietary patterns, nutritional assessment scales and nutrition-related laboratory variables were collected. Binary logistics regression analyses and receiver operating characteristic (ROC) curves were used to indicate the association between nutrition-related variables and AD at different stages.

**Results:**

Totals of 266 subjects, including 73 subjects with NC, 72 subjects with mild cognitive impairment due to AD (AD-MCI) and 121 subjects with dementia due to AD (AD-D) were included. There was no significant difference in dietary patterns, including Mediterranean diet and Mediterranean-DASH diet intervention for neurodegenerative delay (MIND) diet between the three groups. Lower BMI value, smaller hip and calf circumferences, lower Mini Nutritional Assessment (MNA) and Geriatric Nutritional Risk Index (GNRI) scores, and lower levels of total protein, albumin, globulin, and apolipoprotein A1 were associated with AD (all *p* < 0.05). Total protein and albumin levels had the greatest ability to distinguish AD from non-AD (AUC 0.80, 95% CI 0.74–0.84, *p* < 0.001), increased by combining calf circumference, MNA score and albumin level (AUC 0.83, 95% CI 0.77–0.88, *p* < 0.001). Albumin level had the greatest ability to distinguish NC from AD-MCI (AUC 0.75, 95% CI 0.67–0.82, *p* < 0.001), and MNA score greatest ability to distinguish AD-MCI from AD-D (AUC 0.72, 95% CI 0.65–0.78, *p* < 0.001).

**Conclusion:**

Nutritional status of AD patients is significantly compromised compared with normal controls, and tends to be worsened with AD progresses. Early identification and intervention of individuals with nutritional risk or malnutrition may be significantly beneficial for reducing the risk, development, and progression of AD.

## Introduction

As the global population ages, the prevalence of individuals suffering from dementia has risen steeply. Among the cognitive disorders affecting old adults, Alzheimer’s disease (AD) stands as the most prevalent (1). Unfortunately, currently pharmacological interventions have been proven incurable so far. At present, the recognized factors that contribute to AD encompass age, gender, *apolipoprotein E (APOE)* ε4 genotype, and living alone (1). It is therefore paramount to identify and intervene early on the potential risk factors that may promote the onset and progression of AD (2).

According to the recommendations from European Society for Clinical Nutrition and Metabolism (ESPEN), malnutrition is the most common nutritional issue in dementia patients (3). Malnutrition has been linked to impaired cognition, accelerated progression, and increased mortality in AD patients according to prior research (4–6). For example, one population-based study revealed that weight loss was significant in both MCI and dementia patients, potentially serving as an initial indicator of cognitive decline (7). Another study indicated that body mass index (BMI) was substantially decreased in AD patients at the dementia stage compared to those at the mild cognitive impairment (MCI) stage (8), probably serving as a useful predictor of disease progression.

In addition to weight loss and BMI, there existed several variables that might evaluate the nutritional status of individuals, including body composition, dietary patterns, nutritional assessment scales, and peripheral blood laboratory parameters, like albumin, homocysteine, and B vitamins (6, 9–11). Currently, however, there is a paucity of research that has comprehensively evaluated the nutritional status of AD patients, and the predictive values of nutritional variables for AD at different stages remain unclear.

Considering that nutrition is a modifiable risk factor for AD, the aims of this study were to holistically evaluate the nutritional status of AD patients, and determine the potential association of a range of nutritional indices for AD at different stages.

## Materials and methods

### Study population

AD patients were consecutively enrolled from the Center for Cognitive Neurology, Department of Neurology, Beijing Tiantan Hospital, Capital Medical University between April 2019 and April 2023. Patients with MCI due to AD (AD-MCI) and dementia due to AD (AD-D) were diagnosed according to the National Institute of Aging and Alzheimer’s Association (NIA-AA) criteria (12, 13). The age-matched old adults with normal cognition (NC) were enrolled from community at the same time.

The subjects meeting the following criteria were excluded from this study: (1) subjects with neurological diseases that might affect cognition apart from AD, including cerebrovascular diseases, Lewy bodies disease, Parkinson’s disease, corticobasal degeneration, frontotemporal degeneration, primary progressive aphasia, multiple sclerosis, hydrocephalus, limbic encephalitis; (2) subjects with the following conditions leading to malnutrition, including hematological tumors, liver cirrhosis, severe systemic disease, or subtotal gastrectomy; (3) subjects were unable to cooperate with all the examinations for various reasons.

**Figure 1:**
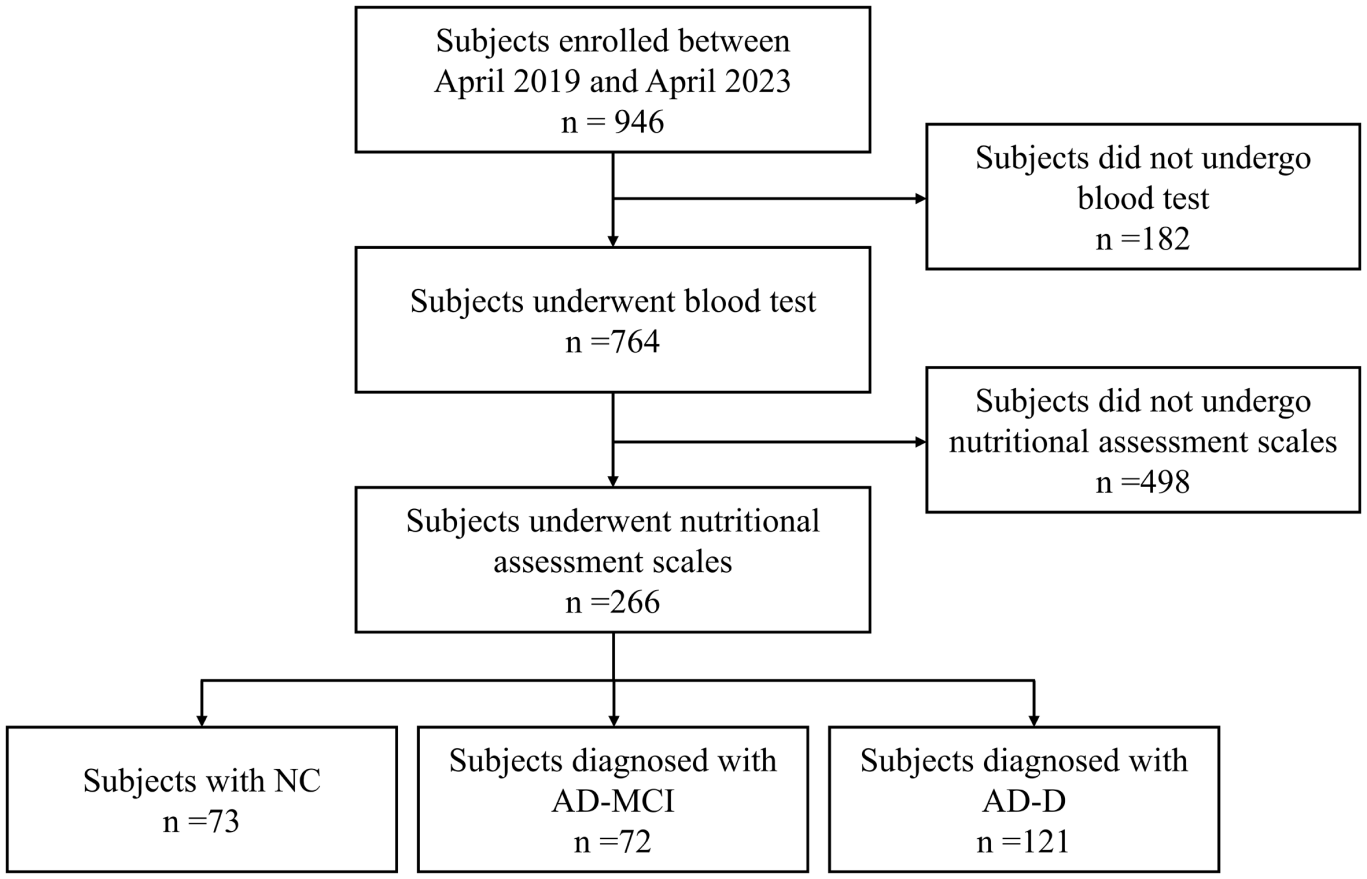
Study flow chart. NC, normal cognition; MCI, mild cognitive impairment; AD, Alzheimer’s disease.

A total of 946 subjects were consecutively enrolled in this study, out of which 182 subjects who did not undergo blood test and 498 subjects who did not undergo assessments of nutritional scales were excluded. Ultimately, 266 subjects were included in this study, with 73 subjects in NC group, 72 subjects in AD-MCI group, and 121 subjects in AD-D group (Figure 1).

### Demographic information

Demographic information, encompassing sex, age, age of onset, years of education, living condition, *APOE* genotype, history of comorbidities, like hypertension, hyperlipidemia, diabetes mellitus, hyperhomocysteinemia, myocardial infarction and atrial fibrillation, and history of medications for treating cognitive impairment and psychotropic disorders, and vitamins, were recorded for all subjects.

### Body composition

Body composition containing the following aspects according to Global Leadership Initiative on Malnutrition (GLIM) criteria (14) were assessed for all subjects.

*Reduced food intake*: reduced food intake within the past 3 months due to multiple reasons, such as medication side effects, dysphagia, poor oral health, gastrointestinal complaints, depression, anorexia, and inadequate nutrition support, was categorized as 25–50%, 50–75%, or ≥75% according to the percentage of reduced food intake in normal requirement.

*Non-volitional weight loss*: non-volitional weight loss was defined as a self-reported body weight loss within the past 6 months, which was categorized as <5%, 5–10%, and >10% based on the percentage of weight loss in original body weight.

*BMI*: BMI was calculated by dividing the measured body weight by the squared measured height (kg/m^2^), and was classified as underweight (BMI < 18.5 kg/m^2^), normal weight (BMI 18.5–23.9 kg/m^2^), overweight (BMI 24.0–27.9 kg/m^2^), and obese (BMI ≥ 28.0 kg/m^2^) according to Chinese Adults Classification Standard (15, 16).

*Muscle mass*: arm, waist, hip and calf circumferences were measured to indirectly evaluate muscle mass of subjects. Waist circumference was measured at the narrowest point of trunk between ribs and upper part of the hip bone. Hip circumference was measured at the widest point of hip and buttocks. Arm circumference was measured at the midpoint between acromion and olecranon processes. Calf circumference was measured at the location where calf was the thickest (17).

### Dietary patterns

Dietary patterns of subjects, including alternate Mediterranean diet (18) and Mediterranean-DASH diet intervention for neurodegenerative delay (MIND) diet (19), were collected to assess the habitual dietary intake of subjects. Alternate Mediterranean diet contains 11 food components, of which 7 are recommended (non-refined cereals, vegetables, fruits, legumes, fish, potatoes and olive oil) and 4 are not recommended (red meat and products, poultry, full-fat dairy products and alcoholic beverages). A score ranging from 0–5 was assigned to each food component, which indicates how often subjects eat each food component in each month, with higher score indicating higher frequency. The total score of the alternate Mediterranean diet ranges from 0–55, with higher score reflecting better adherence.

MIND diet contains 15 food components, among which 10 are good for mental health (green leafy vegetables, other vegetables, nuts, berries, beans, whole grains, seafood, poultry, olive oil and wine), and 5 are unhealthy for mental health (red meats, butter and stick margarine, cheese, pastries and sweets, and fried/fast food). Olive oil consumption was scored 1 if identified by subjects as the primary oil usually used at home and 0 otherwise. A point was assigned to subjects reporting the use of olive oil in cooking every day. For each other food component, 0 was assigned if subjects did not adhere to the recommended diet frequency, 0.5 for moderate adherence, and 1 for good adherence. Total MIND score was calculated by summing up the scores of 15 components. The total score ranges from 0–15, with higher score reflecting richer dietary intake. Details regarding the two dietary scores were added in the Supplementary material.

### Evaluation of nutritional status by nutritional assessment scales

The nutritional status of subjects was assessed using a variety of assessment scales, including Malnutrition Universal Screening Tool (MUST) (20), Nutritional Risk Screening 2002 (NRS 2002) (9), Mini-Nutritional Assessment-short form (MNA-SF) (21), MNA (10), and Geriatric Nutritional Risk Index (GNRI) (22). Detailed descriptions of these nutritional assessment scales were given in the Supplementary material.

### Measurements of nutrition-related laboratory variables in blood

Venous blood samples were collected from the median elbow of subjects under fasting condition on the next morning after admission, and then sent to Department of Laboratory of our hospital for the measuring nutrition-related laboratory variables.

A host of laboratory variables related to nutrition, including hemoglobin A1c (HbA1c), fasting blood glucose (FBG), hemoglobin, blood urea nitrogen (BUN), creatinine, total calcium, total protein, albumin, globulin, albumin/globulin (A/G), prealbumin, triglyceride, total cholesterol, high density lipoprotein cholesterol (HDLC), low density lipoprotein cholesterol (LDLC), apolipoprotein A1, apolipoprotein B, homocysteine, folic acid, vitamin B_12_ and ferritin, were collected to evaluate the nutritional status of subjects.

### Statistical analysis

All analyses were conducted using SPSS Statistics 25.0 (IBM Corporation, New York, United States). Statistical significance was defined as a two-sided *p* < 0.05.

Normality of distributions was assessed using Shapiro–Wilk test. Normally distributed data were displayed as mean ± standard deviation (SD) and compared using the analysis of variance (ANOVA). Skewed data were displayed as median (interquartile range) and compared using Kruskal–Wallis test. Categorical data were displayed as number (percentage) and compared using *χ*^2^-tests. The Kruskal–Wallis test was applied for multiple comparisons in the case of skewed data, while Bonferroni correction was applied for multiple comparisons in the case of categorical data.

Binary logistics regression analyses were conducted to estimate the effect size of the association between nutrition-related variables and AD at different stages, including AD-MCI and AD-D, and presented with the forest diagrams. Key covariates included sex, age, age of onset, years of education, hyperlipidemia, diabetes mellitus and history of medication for treating cognitive impairment. Receiver operating characteristic (ROC) curves were further drawn, and area under the curve (AUC), Youden index, cut-off value, sensitivity, specificity, positive predictive value (PPV) and negative predictive value (NPV) were calculated to estimate the potential ability of nutrition-related variables to distinguish AD from non-AD, NC from AD-MCI, and AD-MCI from AD-D.

## Results

### Clinical characteristics of subjects

The clinical characteristics of NC, AD-MCI, and AD-D groups were presented in Table 1. Among these subjects, 57.14% were female, their average age was 64.89 ± 9.36 years old, their average years of education was 11.75 ± 4.26 years, and 36.84% of cases carried *APOE* ε4 allele. AD-D group had a fewer years of education than NC group (*p* < 0.001). A lower proportion of AD-D group had a history of hyperlipidemia (*p* < 0.001) and diabetes mellitus (*p* = 0.022) compared to NC and AD-MCI groups, and a higher proportion of AD-D group took medications for treating cognitive impairment compared to NC and AD-MCI groups (*p* < 0.001). Furthermore, a lower proportion of AD-D group had a history of diabetes mellitus compared to AD-MCI group (*p* = 0.005).

**Table 1:** Demographic information and nutritional status of NC, AD-MCI, and AD-D groups.

	NC group (*n* = 73)	AD-MCI group (*n* = 72)	AD-D group (*n* = 121)	*p*-value
Demographic information				
Female (*n*, %)	38 (52.05)	42 (58.33)	72 (59.50)	0.644
Age (years, mean ± SD)	63.42 ± 8.49	65.43 ± 7.96	65.45 ± 10.53	0.683
Age of onset (years, mean ± SD)	56.18 ± 13.26	61.15 ± 12.70	60.99 ± 11.64	0.061
Years of education (years, mean ± SD)	13.33 ± 3.99	12.03 ± 3.39	10.61 ± 4.58^b^	<0.001**
Living alone (*n*, %)	3 (4.11)	8 (11.11)	4 (3.31)	0.082
*APOE* ε4 allele carrier (*n*, %)	12 (16.44)	30 (41.67)	47 (38.84)	0.119
History of diseases				
Hypertension (*n*, %)	40 (54.79)	39 (54.17)	51 (42.15)	0.134
Hyperlipidemia (*n*, %)	35 (47.95)	33 (45.83)	20 (16.53)^b^^,^^d^	<0.001**
Diabetes mellitus (*n*, %)	16 (21.92)	23 (31.94)	18 (14.88)^d^	0.022*
Hyperhomocysteinemia (*n*, %)	2 (2.74)	0 (0.00)	4 (3.31)	0.143
Myocardial infarction (*n*, %)	3 (4.11)	0 (0.00)	2 (1.65)	0.120
Atrial fibrillation (*n*, %)	1 (1.37)	1 (1.39)	4 (3.31)	0.572
Medications				
Anti-cognitive impairment drugs (*n*, %)	2 (2.74)	5 (6.94)	46 (38.02)^b^^,^^d^	<0.001**
Anti-psychotropic drugs (*n*, %)	2 (2.74)	4 (5.55)	5 (4.13)	0.624
Vitamins (*n*, %)	3 (4.11)	2 (2.78)	0 (0.00)	0.089
Smoking (*n*, %)	14 (19.18)	18 (25.00)	32 (26.45)	0.506
Drinking (*n*, %)	21 (28.77)	21 (29.17)	24 (19.83)	0.229
Body composition				
Reduced food intake within the past 3 months				0.543
None (*n*, %)	63 (86.30)	57 (79.17)	91 (75.21)	
25–50% (*n*, %)	5 (6.85)	9 (12.50)	13 (10.74)	
50–75% (*n*, %)	4 (5.48)	4 (5.55)	13 (10.74)	
≥75% (*n*, %)	1 (1.37)	2 (2.78)	4 (3.31)	
Body weight loss within the past 6 months				0.880
<5% (*n*, %)	66 (90.41)	65 (90.28)	110 (90.91)	
5–10% (*n*, %)	5 (6.85)	6 (8.33)	10 (8.26)	
>10% (*n*, %)	2 (2.74)	1 (1.39)	1 (0.83)	
BMI (kg/m^2^, mean ± SD)	25.27 ± 3.37	24.77 ± 3.38	23.20 ± 3.29^b^^,^^d^	<0.001**
Underweight (*n*, %)	1 (1.37)	1 (1.39)	5 (4.13)	
Normal weight (*n*, %)	26 (35.62)	34 (47.22)	71 (58.68)	
Overweight (*n*, %)	30 (41.10)	28 (38.89)	36 (29.75)	
Obese (*n*, %)	16 (21.92)	9 (12.50)	9 (7.44)	
Circumference				
Arm circumference (cm, mean ± SD)	28.65 ± 2.38	28.11 ± 4.30	27.42 ± 4.69^b^	0.026*
Waist circumference (cm, mean ± SD)	88.38 ± 8.66	89.07 ± 9.02	83.49 ± 13.98^a^^,^^d^	0.007**
Hip circumference (cm, mean ± SD)	99.93 ± 6.23	98.05 ± 5.77	96.51 ± 9.26^b^	0.008**
Calf circumference (cm, mean ± SD)	36.46 ± 2.62	36.05 ± 3.57	34.04 ± 3.65^b^^,^^d^	<0.001**
Dietary patterns				
Mediterranean diet (points, mean ± SD)	27.98 ± 6.40	27.52 ± 5.74	27.18 ± 6.01	0.758
MIND diet [points, median (quartile)]	10.00 (9.00, 10.00)	9.00 (8.00, 10.00)	9.00 (8.00, 10.00)	0.328
Nutritional assessment scales				
MUST [points, median (quartile)]	0.00 (0.00, 0.00)	0.00 (0.00, 0.00)	0.00 (0.00, 0.00)	0.488
NRS2002 [points, median (quartile)]	0.00 (0.00, 1.00)	0.00 (0.00, 1.00)	0.00 (0.00, 1.00)	0.996
MNA-SF [points, median (quartile)]	13.00 (12.00, 14.00)	13.00 (12.00, 14.00)	12.00 (11.00, 13.00)^b^^,^^d^	<0.001**
MNA [points, median (quartile)]	26.00 (24.25, 27.00)	25.00 (23.13, 26.50)	23.00 (21.00, 25.00)^b^^,^^d^	<0.001**
GNRI (points, mean ± SD)	106 ± 8.11	103 ± 6.83	101 ± 8.16^b^^,^^d^	<0.001**
Nutrition-related laboratory variables				
HbA1C [%, median (quartile)]	5.90 (5.60, 6.45)	5.90 (5.60, 6.50)	5.90 (5.60, 6.30)	0.834
FBG [mmol/L, median (quartile)]	5.09 (4.65, 5.83)	4.97 (4.57, 5.92)	4.84 (4.47, 5.60)	0.162
Hemoglobin [g/L, mean ± SD)]	136 ± 11.70	131 ± 13.66	132 ± 13.92	0.392
BUN [mmol/L, median (quartile)]	5.50 (4.65, 6.50)	6.00 (4.90, 7.00)	5.50 (4.63, 6.50)	0.135
Creatinine [μmol/L, median (quartile)]	60.00 (53.80, 69.15)	60.20 (51.30, 68.80)	58.30 (51.18, 67.25)	0.303
Total calcium [mmol/L, median (quartile)]	2.38 (2.30, 2.45)	2.33 (2.29, 2.39)^b^	2.30 (2.23, 2.37)^b^	<0.001**
Total protein [g/L, median (quartile)]	71.20 (67.80, 73.95)	65.70 (62.30, 71.00)^b^	64.15 (61.50, 67.25)^b^^,^^c^	<0.001**
Albumin [g/L, median (quartile)]	43.90 (42.00, 45.30)	39.70 (38.30, 43.20)^b^	39.30 (37.60, 41.30)^b^	<0.001**
Globulin [g/L, median (quartile)]	27.90 (25.10, 29.50)	26.00 (23.90, 28.00)^b^	25.10 (23.50, 27.30)^b^	<0.001**
A/G [median (quartile)]	1.60 (1.50, 1.70)	1.60 (1.40, 1.70)	1.55 (1.40, 1.70)	0.715
Prealbumin (g/L, mean ± SD)	243 ± 39.28	241 ± 45.10	245 ± 43.93	0.860
Triglyceride [mmol/L, median (quartile)]	1.23 (0.90, 1.64)	1.14 (0.88, 1.50)	1.00 (0.73, 1.43)	0.059
Total cholesterol [mmol/L, median (quartile)]	4.67 (3.95, 5.22)	4.55 (3.87, 5.27)	4.45 (3.81, 5.16)	0.685
HDLC [mmol/L, median (quartile)]	1.52 (1.21, 1.73)	1.44 (1.21, 1.63)	1.38 (1.21, 1.64)	0.192
LDLC (mmol/L, mean ± SD)	2.72 ± 0.83	2.73 ± 0.95	2.67 ± 0.86	0.884
Apolipoprotein A1 (g/L, mean ± SD)	1.62 ± 0.29	1.47 ± 0.24^b^	1.41 ± 0.24^b^	<0.001**
Apolipoprotein B [g/L, median (quartile)]	0.80 (0.66, 0.92)	0.82 (0.69, 0.95)	0.78 (0.66, 0.93)	0.469
Homocysteine [μmol/L, median (quartile)]	11.20 (9.70, 13.16)	10.94 (9.01, 12.98)	12.64 (10.60, 14.89)^b^^,^^d^	<0.001**
Folic acid [ng/mL, median (quartile)]	9.49 (5.84, 13.18)	8.76 (5.62, 14.06)	5.35 (4.04, 9.07)^b^^,^^d^	0.001**
Vitamin B_12_ [pg/mL, median (quartile)]	502 (305, 600)	465 (344, 637)	351 (265, 480)^c^	0.005**
Ferritin [ng/mL, median (quartile)]	85.80 (26.53, 110)	88.20 (57.20, 148)	89.35 (58.88, 154)	0.476

Data were presented as number (percentage), means ± SD or median (quartile). NC, normal cognition; MCI, mild cognitive impairment; AD, Alzheimer’s disease; APOE, apolipoprotein E; BMI, body mass index; Medi, Mediterranean diet; MIND, Mediterranean-DASH intervention for neurodegenerative delay diet; MUST, malnutrition universal screening tool; NRS2002, the nutritional risk screening 2002; MAN-SF, the mini nutritional assessment-short form; MNA, the mini nutritional assessment; GNRI, geriatric nutrition risk index; FBG, fasting blood glucose; BUN, blood urea nitrogen; A/G, albumin/globulin; HDLC, high density lipoprotein cholesterol; LDLC, low density lipoprotein cholesterol. *p < 0.05, **p < 0.01.

a *p* < 0.05 compared with NC group upon post-hoc testing.

b *p* < 0.01 compared with NC group upon post-hoc testing.

c *p* < 0.05 compared with AD-MCI group upon post-hoc testing.

d *p* < 0.01 compared with AD-MCI group upon post-hoc testing.

Regarding body composition, AD-D group had lower arm and hip circumferences than NC group, and had lower BMI, waist and calf circumferences compared to NC and AD-MCI groups (all *p* < 0.05).

Comparing dietary patterns among NC, AD-MCI, and AD-D groups, there were no statistically significant differences in the Mediterranean diet and MIND scores between the three groups (all *p* < 0.05). It was found that the scores of Mediterranean diet (*p* = 0.758) and MIND diet (*p* = 0.328) of AD-D group were slightly lower than NC and AD-MCI groups.

Regarding nutritional assessment scales, AD-D group had lower scores of MNA-SF and MNA than NC and AD-MCI groups (all *p* < 0.001). Both AD-MCI (*p* < 0.001) and AD-D (*p* = 0.003) groups had lower GNRI scores than NC group.

In terms of nutrition-related laboratory variables, AD subjects had lower levels of total calcium, total protein, albumin, globulin, apolipoprotein A1 and folic acid, and higher homocysteine level than NC subjects (all *p* < 0.05). In AD subjects, AD-D group had lower levels of total protein (*p* = 0.023), folic acid (*p* = 0.003) and vitamin B_12_ (*p* = 0.010), and higher homocysteine level in blood (*p* = 0.001) than AD-MCI group.

### The association between nutrition-related variables and AD

**Figure 2:**
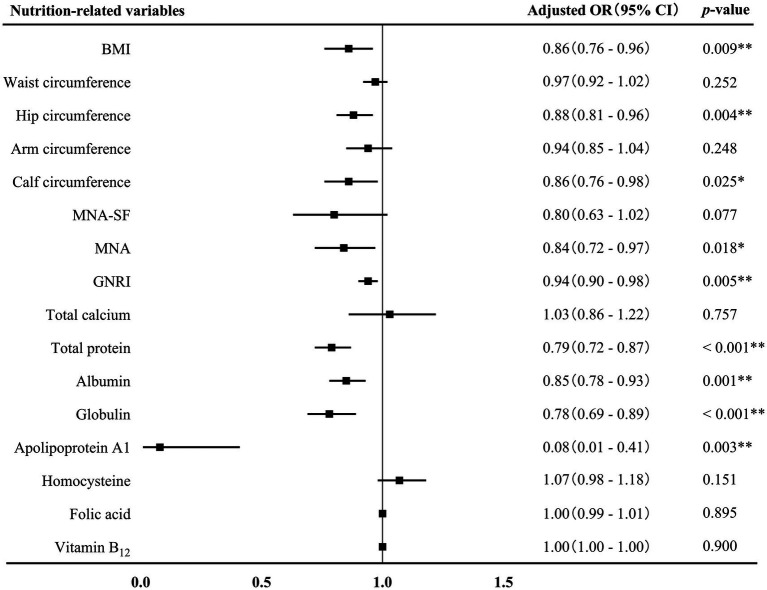
Association between nutrition-related variables and AD. AD, Alzheimer’s disease; OR, odds ratio; CI, confidence interval; BMI, body mass index; MAN-SF, mini nutritional assessment-short form; MNA, mini nutritional assessment; GNRI, geriatric nutrition risk index. ^*^p < 0.05 and ^**^p < 0.01.

Binary logistics regression analyses were conducted to investigate the association between nutrition-related variables and AD (Figure 2). BMI (adjusted OR 0.86, 95% CI 0.76–0.96, *p* = 0.009), hip circumference (adjusted OR 0.88, 95% CI 0.81–0.96, *p* = 0.004), calf circumference (adjusted OR 0.86, 95% CI 0.76–0.98, *p* = 0.025), MNA score (adjusted OR 0.84, 95% CI 0.72–0.97, *p* = 0.018), GNRI score (adjusted OR 0.94, 95% CI 0.90–0.98, *p* = 0.005), and levels of total protein (adjusted OR 0.79, 95% CI 0.72–0.87, *p* < 0.001), albumin (adjusted OR 0.85, 95% CI 0.78–0.93, *p* = 0.001), globulin (adjusted OR 0.78, 95% CI 0.69–0.89, *p* < 0.001), and apolipoprotein A1 (adjusted OR 0.08, 95% CI 0.01–0.41, *p* = 0.003) were independently and negatively associated with AD after adjusting for sex, age, age of onset, years of education, hyperlipidemia, diabetes mellitus and history of medications for treating cognitive impairment.

**Figure 3:**
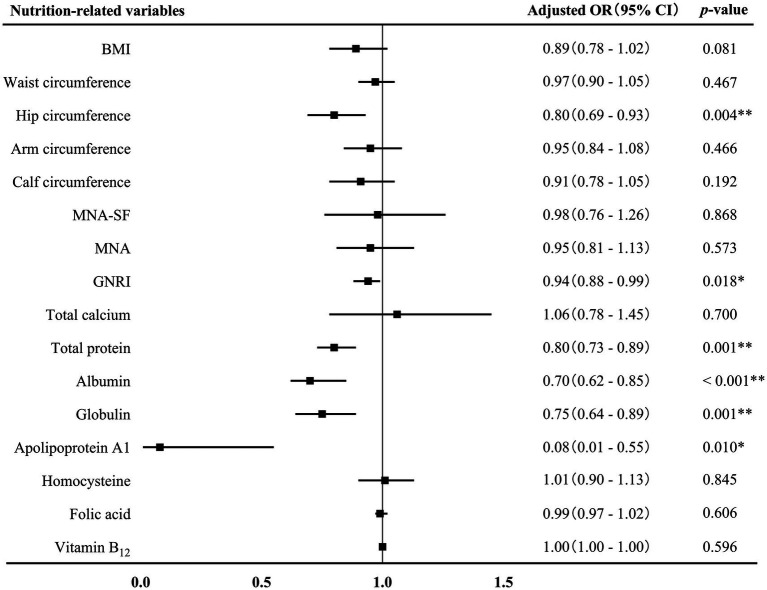
Association between nutrition-related variables and AD-MCI. AD, Alzheimer’s disease; MCI, mild cognitive impairment; OR, odds ratio; CI, confidence interval; BMI, body mass index; MAN-SF, mini nutritional assessment-short form; MNA, mini nutritional assessment; GNRI, geriatric nutrition risk index. ^*^p < 0.05 and ^**^p < 0.01.

**Figure 4:**
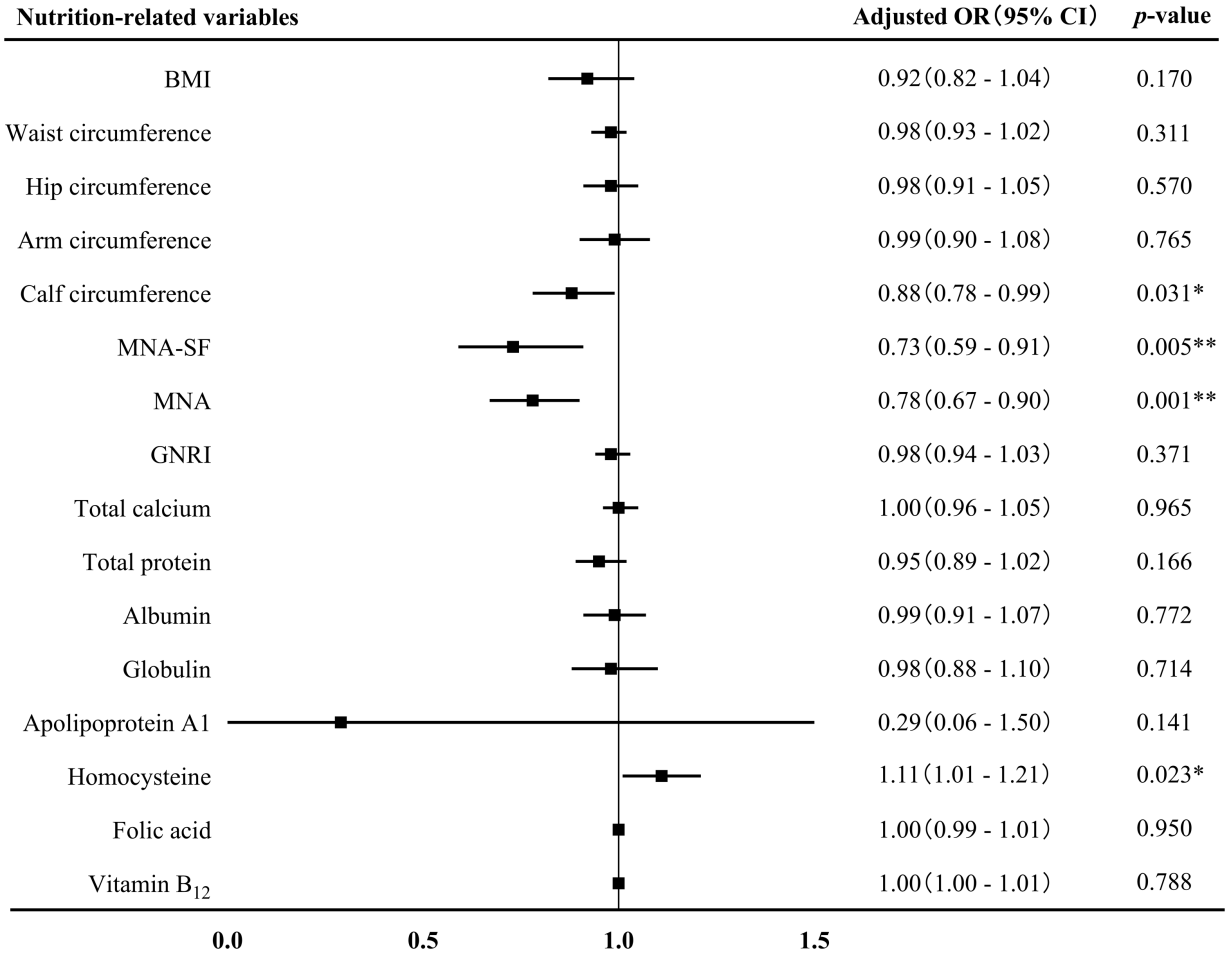
Association between nutrition-related variables and AD-D. AD, Alzheimer’s disease; OR, odds ratio; CI, confidence interval; BMI, body mass index; MAN-SF, mini nutritional assessment-short form; MNA, mini nutritional assessment; GNRI, geriatric nutrition risk index. ^*^p < 0.05 and ^**^p < 0.01.

The associations between nutrition-related variables and AD at different stages were further conducted. First of all, binary logistics regression displayed that GNRI score (adjusted OR 0.94, 95% CI 0.88–0.99, *p* = 0.018), hip circumference (adjusted OR 0.80, 95% CI 0.69–0.93, *p* = 0.004) and the levels of total protein (adjusted OR 0.80, 95% CI 0.73–0.89, *p* = 0.001), albumin (adjusted OR 0.70, 95% CI 0.62–0.85, *p* < 0.001), globulin (adjusted OR 0.75, 95% CI 0.64–0.89, *p* = 0.001), and apolipoprotein A1 (adjusted OR 0.08, 95% CI 0.01–0.55, *p* = 0.001) were negatively associated with AD-MCI (Figure 3). These associations remained significant after adjusting for sex, age, age of onset, years of education, hyperlipidemia, diabetes mellitus and history of medications for treating cognitive impairment. In addition, binary logistics regression analyses presented that MNA-SF score (adjusted OR 0.73, 95% CI 0.59–0.91, *p* = 0.005), MNA score (adjusted OR 0.78, 95% CI 0.67–0.90, *p* = 0.001), and calf circumference (adjusted OR 0.88, 95% CI 0.78–0.99, *p* = 0.031) were independently and negatively associated with AD-D, while homocysteine level (adjusted OR 1.11, 95% CI 1.01–1.21, *p* = 0.023) was positively associated with AD-D after adjusting for the above confounding factors (Figure 4).

### Potential diagnostic ability of nutrition-related variables for AD

**Figure 5:**
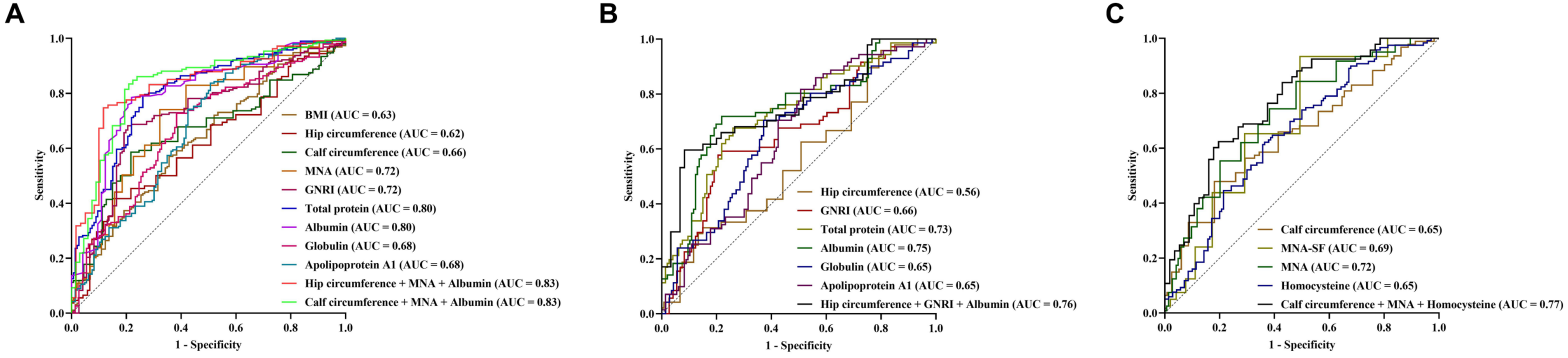
Predictive value of nutrition-related variables for AD **(A)**, AD-MCI **(B)**, and AD-D **(C)**. AD, Alzheimer’s disease; MCI, mild cognitive impairment; BMI, body mass index; AUC, area under the curve; MNA, mini nutritional assessment; GNRI, geriatric nutrition risk index; MAN-SF, mini nutritional assessment-short form.

First, ROC curves were used to assess the ability of nutrition-related variables to distinguish AD from non-AD. According to the ROC curve displayed, the maximum AUC related to AD was observed for the levels of total protein and albumin (AUC 0.80, 95% CI 0.74–0.84, *p* < 0.001), the identified cut-off values for total protein and albumin were 68.60 g/L and 41.90 g/L, respectively. In distinguishing AD from non-AD, the sensitivity, specificity, PPV and NPV of total protein level were 79.06%, 73.97%, 88.80%, and 57.51%, respectively, and of albumin level were 78.35%, 78.08%, 90.34%, and 58.22%, respectively. The combination MNA score, calf circumference and albumin level exhibited a high AUC (AUC 0.83, 95% CI 0.77–0.88, *p* < 0.001), which sensitivity, specificity, PPV and NPV were 83.44%, 79.10%, 90.01%, and 67.91%, respectively (Figure 5A and Supplementary Table S1).

Next, the ability of nutrition-related variables to distinguish NC from AD-MCI were investigated. NC and AD-MCI groups were selected, and ROC curve indicated that albumin level had the maximum AUC for distinguish NC from AD-MCI (AUC 0.75, 95% CI 0.67–0.82, *p* < 0.001), with a cut-off value of 41.90 g/L, and sensitivity, specificity, PPV and NPV of 72.86%, 78.08%, 76.12%, and 75.00%, respectively. The combination of GNRI score, hip circumference, and albumin level increased AUC (AUC 0.76, 95% CI 0.66–0.83, *p* < 0.001), with sensitivity, specificity, PPV and NPV of 59.57%, 91.67%, 84.86%, and 74.32%, respectively (Figure 5B and Supplementary Table S2).

Finally, the ability of nutrition-related variables to distinguish AD-MCI from AD-D were investigated. AD-MCI and AD-D groups were selected, and ROC curve suggested that MNA score had the maximum AUC for distinguish AD-MCI from AD-D (AUC 0.72, 95% CI 0.65–0.78, *p* < 0.001), with cut-off value of 24.50 points, and the sensitivity, specificity, PPV and NPV of 74.38%, 61.11%, 76.28%, and 58.66%, respectively. The combination of MNA score, calf circumference and homocysteine level exhibited a high AUC (AUC 0.77, 95% CI 0.69–0.83, *p* < 0.001), with sensitivity, specificity, PPV and NPV of 62.37%, 80.36%, 84.05%, and 56.27%, respectively (Figure 5C and Supplementary Table S3).

## Discussion

This study comprehensively evaluated the relationship between a variety of nutrition-related variables and the occurrence and progression of AD. The data revealed that a variety of nutrition-related variables, including nutritional assessment scales, body composition, and nutrition-related laboratory indicators, might be independently associated with the occurrence and progression of AD. These nutritional variables might have the potential to distinguish AD from non-AD, NC from AD-MCI, and AD-MCI from AD-D. The combined variables significantly improved their distinguish ability.

As malnutrition is a common issue in AD, and associated with the enhanced incidence, accelerated disease progression and increased mortality of AD, thus, the nutritional status of each patient needs to be seriously taken into consideration (3, 5). The results of this study exhibited that the nutritional status of AD patients was worse than that of age-matched cognitively normal individuals, and the nutritional status further deteriorated with disease progression. Currently, the underlying causes of malnutrition in AD remain incompletely understood. It was suggested that the factors affecting food intake, such as taste disorders, olfactory dysfunction and compromised appetite, were the primary contributors to the malnutrition in the early stage of AD (23, 24). As disease progresses, chronic inflammatory response was gradually intensified, resulting in excessive protein and energy consumptions as the main cause of malnutrition at the middle and late stages of AD (25, 26). Moreover, neuropsychiatric symptoms, eating disorder and dysphagia in the later stage of AD further exacerbated malnutrition (27). It was hypothesized that an increased nutritional supplementation could facilitate the activation of neural protein synthesis, and increase the production of new cortical connections and axonal sprouting, and thereby showing a beneficial effect on cognitive recovery (28).

### The association between body composition and AD

In the present study, body composition of subjects was evaluated in several ways, including reduced food intake, non-volitional weight loss, BMI, and circumferences of arm, waist, hip, and calf. We found that a lower BMI was independently associated with the occurrence of AD, corroborating earlier research findings (7). The association between BMI and dementia has been a subject with much debate. U-shaped or J-shaped associations were found between BMI and physical functioning. Specifically, both too low and too high BMI were associated with poor physical function (29). It was previously reported that low BMI increased the risk of AD and disability in activities of daily living (30). It is well recognized that BMI is a measure of muscle mass rather than body fatness in older adults because of the changes in skeletal muscle and abdominal fat with aging (31). Low BMI may denote a predominance of muscle mass attenuation, leading to malnutrition and high risk of AD (32).

This study also uncovered that AD patients at the dementia stage had reduced arm, waist, hip, and calf circumferences in comparison to those at the MCI and NC stages. Notably, hip circumference < 94.50 cm contributed to distinguish NC from AD-MCI, and calf circumference < 33.50 cm contributed to distinguish AD-MCI from AD-D. Prospective cohort studies revealed that small arm and calf circumferences increased disease severity and mortality in AD patients (33, 34). Additionally, a smaller waist circumference was linked to lower level of β amyloid protein (Aβ)_1-42_ and higher levels of phosphorylated tau (P-tau) and total tau (T-tau) in the cerebrospinal fluid of AD patients (6, 35). This phenomenon may be attributed to the utilization of skeletal muscle mass as a nutritional reservoir in response to a prolonged state of negative energy balance during disease (32). Consequently, the muscle mass of AD patients gradually decreases as the disease progresses in AD.

More interestingly, there is an accumulating body of evidence indicating that hormone may play a role in the effect of BMI and muscle mass in AD. Studies have revealed that AD patients have decreased blood levels of sex hormones such as estrogen and testosterone (36, 37). Estradiol has been shown to improve cognition by mediating synaptic plasticity and increasing dendritic spine density (37), while testosterone has been shown to improve cognition by stimulating microglial phagocytosis, inhibiting neuroinflammation, and reducing Aβ deposition (38). A recent study found a significantly positive association between estrogen level and BMI in AD patients (39). It is well known that BMI is positively correlated with serum estrogen levels in postmenopausal women because of the high abundance of aromatase in adipose tissue (40). Estrogen and testosterone have a steroidal structure and are highly lipophilic. Serum steroids are readily transferred and accumulated in the lipid-rich brain. Therefore, we believe that the reduction of BMI may affect the occurrence and progression of AD by reducing the levels of estrogen and testosterone.

In addition to sex hormones, insulin may also play an important role in the relationship between malnutrition and AD. Insulin affects cell growth and differentiation, protein synthesis, and inhibits catabolic processes, such as glycolysis, lipolysis and proteolysis. In addition, insulin can promote the growth of neurons, regulate the synthesis and uptake of neurotransmitters, and improve cognitive function (41). Insulin resistance has been demonstrated to be prevalent in AD (41). A recent Mendelian randomization study found that sarcopenia led to AD and insulin resistance plays a mediating role (42).

### The association between dietary pattern and AD

In this study, there was no association was found between dietary patterns, such as Mediterranean diet as well as MIND diet, and AD. So far, the association between these two dietary patterns and AD remains controversial. Several studies demonstrated that Mediterranean diet and MIND diet contributed to the cognitive function of AD patients as well as old adults (43, 44). For example, a parallel-group randomized clinical trial of 447 cognitively healthy subjects from Spain (mean age 66.9 years) found that Mediterranean diet improved cognitive function of subjects (45). A prospective cohort study involving 923 cognitively healthy subjects aged 58 to 98 years from America observed that adherence to MIND diet for 4.5 years reduced the risk of AD by 53% (43). High adherence to these diets was associated with a decreased risk of cognitive impairment, AD-MCI, and AD-D, as well as the transition from AD-MCI to AD-D (46). The potential neuroprotective mechanisms of these diets might be due to their ability to reduce the levels of inflammation and oxidative stress in individuals (47). However, other studies reported no protective effects of these diets (48–50). A prospective cohort study from America involving 1,528 cognitively healthy subjects aged 60 to 64 years showed that adherence to Mediterranean diet for 4 years failed to delay cognitive decline (48). Moreover, in a recent two-site, randomized, controlled trial from America, 604 subjects aged 65 years or older without cognitive impairment but with a family history of AD and BMI ≥ 25 kg/m^2^ had no significant improvement in cognitive function after 3 years of MIND diet (50). Conflicting results obtained from these studies that evaluated the association between the Mediterranean diet with AD are due to the differences in method and time duration of these studies.

### The association between nutritional assessment scales and AD

Our study was the first one to investigate the relationship between various nutritional assessment scales and AD. We found that lower scores of MNA, MNA-SF, and GNRI were markedly associated with the occurrence and progression of AD.

MNA is a comprehensive and widely used scale to assess early malnutrition in individuals. A previous study revealed that the MNA score of AD patients was significantly lower than that of NC individuals, and it tended to decrease as the disease progressed (6). Our study further found that the MNA score might have the potential to distinguish AD from non-AD, as well as AD-MCI from AD-D.

MNA-SF is a reliable and highly sensitive tool for rapidly screening nutritional status, and has favorable comparability with the full MNA (21). Previous studies reported that AD patients had lower MNA-SF score compared with NC individuals, and the lower MNA-SF score was correlated with the deterioration of psychological symptoms of AD, particularly verbal aggressiveness/emotional disinhibition (51, 52). We revealed that the MNA-SF score <13 points contributed to distinguish AD-MCI from AD-D.

Unlike other nutritional assessment scales, GNRI only requires weight, height, and serum albumin level, and its score can be calculated without patient’s cooperation or a nutritional specialist, thereby guaranteeing the generalizability of both research and clinical use. So far, GNRI score was validated for predicting the outcomes of a variety of diseases, such as stroke, cardiovascular disease, chronic kidney disease, etc. (53–56). Recent studies revealed its association with the development of post-stroke cognitive impairment (53, 54). This association between GNRI score and cognitive function was also found in the Chinese old adults according to a longitudinal cohort study (57). However, there is a lack of study on the GNRI score and AD until now. On the basis, our study was the first one to find a relationship between a lower GNRI score and AD. More specifically, we found that the GNRI score <104 contributed to distinguish AD from non-AD, and NC from AD-MCI, which was helpful to visually monitor the nutritional status of individuals and guide nutritional intervention.

### The association between nutrition-related variables and AD

Reduced levels of peripheral blood proteins are commonly observed as indications of malnutrition. The present study identified significantly decreased total protein, albumin, and globulin levels in the peripheral blood of AD patients compared to NC individuals. Moreover, the lower levels of these proteins were associated with disease severity. Previous investigation revealed the association between the lower levels of total protein and albumin and cognitive impairment in AD patients, and these reduced protein levels were identified as independent risk factors for rapid cognitive decline (58–60). A phase 2b/3 trial suggested that plasma exchange with albumin replacement could slow cognitive and functional decline in AD patients (61). Inadequate protein intake due to some factors, such as dysphagia and decline in appetite, may be one of the primary reasons for the declined protein levels observed in AD patients at advanced stage. Furthermore, the involvement of albumin in AD was attributed to its ability to selectively bind to and transport Aβ, and suppressed the amyloid formation by binding to the oligomeric or polymeric Aβ (62). In addition, albumin possessed antioxidant property and had the capacity to promote the synthesis of neurotrophins, facilitate gliosis, and regulate neuroinflammation (63–65). Other studies have also found the evidence supporting the notion that malnutrition causes thiamine metabolism disorders, affecting the activity and expression of thiamine diphosphate-related enzymes, thus increasing vascular inflammation and impairment of glomerular and tubular structure. This may also be the one of the reasons for the low levels of these proteins in the peripheral blood of AD patients (58). Importantly, this study delved into the potential diagnostic ability of these proteins for AD, and for the first time revealed that total protein level < 68.60 g/L, albumin level < 41.90 g/L, and globulin level < 27.10 g/L contributed to distinguish AD from non-AD. Notably, low levels of albumin and globulin also contributed to distinguish NC from AD-MCI.

Apolipoprotein A1, a prominent variant of high-density lipoprotein synthesized by liver and intestine, plays a crucial role in reversing cholesterol transport. It was reported that apolipoprotein A1 possessed the ability to reduce the accumulation of Aβ, mitigated associated toxicity, prevented brain atrophy, and protected cognitive function (66). Our study demonstrated that plasma apolipoprotein A1 level in AD patients were markedly lower than those in NC individuals (67). Significantly, we further presented that apolipoprotein A1 might be helpful to distinguish NC from AD-MCI, illustrating that apolipoprotein A1 might play a more crucial role at the early stage of AD, and will serve as a target for the early intervention of AD in the future.

B vitamins, including vitamin B_12_ and folic acid, are the indispensable components of mono-carbon metabolism and essential for maintaining cellular methylation capacity (68). Vitamin B_12_ and folic acid deficiencies disrupted one-carbon metabolism, resulting in elevation of homocysteine through reducing enzymatic activities involved in remethylation or transsulfuration processes (69). The results from this study revealed that AD patients exhibited significantly elevated homocysteine level and declined folate and vitamin B_12_ levels compared to NC individuals. The homocysteine level was positively and folate and vitamin B_12_ levels were negatively correlated with disease severity. Specifically, the elevated homocysteine level could distinguish AD-MCI from AD-D. The potential reasons might not only be due to its contribution to vascular damage but also its involvement in oxidative stress (11).

### The diagnostic ability of combined nutrition-related variables for AD

This study further explored the potential diagnostic ability of combining multiple nutrition-related variables for AD. The results showed that the combination of hip and calf circumference, MNA score and albumin level significantly improved the ability to distinguish AD from non-AD. Moreover, the combination of hip circumference, GNRI score and albumin level improved the ability to distinguish NC from AD-MCI, and the combination of calf circumference, MNA score and homocysteine level improved the ability to distinguish AD-MCI from AD-D. These indicators may have the potential to serve as predictive models in future studies. A comprehensive assessment of an individual’s nutritional status may be crucial for recognizing the occurrence and progression of AD.

### Limitations

This study had limitations. Firstly, limited sample size may impact the reliability and applicability of results, which needs to be further validated with large samples in the future. Secondly, more than one evaluator led to the heterogeneity of the measurement results of the scales. Additionally, the cross-sectional nature may inhibit the establishment of causal relationships and observation of dynamic changes in our findings, and introduce memory and selection biases. Efforts will be made to delve deeper into the association between nutritional variables and underlying mechanisms of AD.

## Conclusion

In this study, nutrition-related variables, including body composition, dietary pattern, nutritional assessment scales and nutritional laboratory variables in blood are included to comprehensively evaluate the association between these variables and AD. Multiple nutrition-related variables were significantly associated with the occurrence and progression of AD. Since malnutrition is a risk factor that can be intervened, early identification and intervention of individuals with nutritional risk or malnutrition are significantly beneficial for reducing the risk, development, and progression of AD.

## Data availability statement

The data analyzed in this study are subject to the following licenses/restrictions: the data are available from the first author or the corresponding author upon reasonable request. Requests to access these datasets should be directed to WeZ, ttyyzw@163.com.

## Ethics statement

The studies involving humans were approved by Beijing Tiantan Hospital, Capital Medical University. The studies were conducted in accordance with the local legislation and institutional requirements. The participants provided their written informed consent to participate in this study.

## Author contributions

MH: Conceptualization, Formal analysis, Investigation, Methodology, Writing – original draft. TL: Investigation, Writing – original draft. ZL: Investigation, Visualization, Writing – original draft. JhL: Investigation, Writing – original draft. JQ: Investigation, Writing – original draft. JL: Investigation, Writing – original draft. PG: Investigation, Writing – original draft. YZ: Formal analysis, Writing – review & editing. DL: Investigation, Writing – original draft. HG: Investigation, Writing – original draft. WiZ: Investigation, Writing – original draft. ZZ: Investigation, Writing – original draft. HY: Investigation, Writing – original draft. WnZ: Investigation, Writing – original draft. RW: Investigation, Writing – original draft. FZ: Investigation, Writing – original draft. WeZ: Conceptualization, Project administration, Supervision, Writing – review & editing.

## Glossary

AD: Alzheimer’s disease
APOE: apolipoprotein E
ESPEN: European Society for Clinical Nutrition and Metabolism
BMI: body mass index
MCI: mild cognitive impairment
NC: normal cognition
AD-MCI: MCI due to AD
AD-D: dementia due to AD
NIA-AA: National Institute of Aging and Alzheimer’s Association
GLIM: Global Leadership Initiative on Malnutrition
MIND: Mediterranean-DASH diet intervention for neurodegenerative delay
MUST: Malnutrition Universal Screening Tool
NRS2002: Nutritional Risk Screening 2002
MNA-SF: Mini Nutritional Assessment-short form
MNA: Mini Nutritional Assessment
GNRI: Geriatric Nutritional Risk Index
HbA1c: hemoglobin A1c
FBG: fasting blood glucose
BUN: blood urea nitrogen
A/G: albumin/globulin
HDLC: high density lipoprotein cholesterol
DLC: low density lipoprotein cholesterol
ANOVA: analysis of variance
SD: standard deviation
ROC: receiver operating characteristic
AUC: area under the curve
PPV: positive predictive value
NPV: negative predictive value
OR: odds ratio
CI: confidence interval
Aβ: β amyloid protein
P-tau: phosphorylated tau
T-tau: total tau
