# Supplementary material for: An investigation into the potential association between nutrition and Alzheimer’s disease
Source: Front Nutr. 2024 Mar 7;11:1306226. doi: 10.3389/fnut.2024.1306226 (PMC10955128; doi:10.3389/fnut.2024.1306226)
Supplement: Supplementary file 1 [file Data_Sheet_1.docx]

**An investigation into the potential association between nutrition and Alzheimer’s disease**

Mingyue He^1^, Tenghong Lian^2^, Zhan Liu^1^, Jinghui Li^1^, Jing Qi^1^, Jing Li^1^, Peng Guo^2^, Yanan Zhang^3^, Dongmei Luo^1^, Huiying Guan^1^, Weijia Zhang^1^, Zijing Zheng^1^, Hao Yue^1^, Wenjing Zhang^1^, Ruidan Wang^1^, Fan Zhang^1^, Wei Zhang^1,2,4,5,6^

**Supplementary description of nutritional assessment scales**

MUST(1)

MUST is a 5-step screening tool to identify subjects who are at risk of malnutrition. It is composed of 3 clinical parameters rated as 0, 1 or 2 as follows: BMI > 20.0 kg/m^2^ = 0 point, 18.5-20.0 kg/m^2^ = 1 point, < 18.5 kg/m^2^ = 2 points; weight loss within the last 3-6 months less than 5% = 0 point, 5-10% = 1 point, > 10% = 2 points; presence of acute disease: 2 points are added in the case of acutely ill patients with no nutritional intake or likelihood of no nutritional intake for more than 5 days.

NRS 2002(2)

NRS 2002 encompasses 3 domains: (1) Nutritional parameters. Weight loss > 5% in 3 months or food intake 50%-75% of normal requirements in preceding week = 1 point; weight loss > 5% in 2 months, BMI 18.5-20.5 kg/m^2^, and diminished general condition, or food intake 25%-60% of normal in preceding week = 2 points; and weight loss > 5% in 1 month or > 15% in 3 months, BMI < 18.5 kg/m^2^, and diminished general condition, or food intake 0%-25% of normal in preceding week = 3 points. (2) Severity of disease. Subjects with chronic disease and acute complications, including cirrhosis, chronic obstructive pulmonary disease, chronic hemodialysis, diabetes, and cancer = 1 point; stroke = 2 points; and intensive care patients with Acute Physiology and Chronic Health Evaluation (APACHE) score > 10 = 3 points. (3) Age: Age > 70 years = 1 point. The score ranges from 0 to 7, with score of > 3 points indicating an increasing risk of malnutrition.

MNA-SF(3)

MNA-SF is a simplified version of MNA by identifying a subset of questions from the full MNA that had high sensitivity, specificity, and correlation to the full MNA. The scale involves 6 questions: food intake, weight loss, mobility, psychological stress or acute disease, neuropsychological problems, and BMI/calf circumference. Subjects were categorized as: normal nutritional status (MNA-SF score ≥ 12 points), at risk of malnutrition (MNA-SF score = 8-11 points) and malnourished (MNA-SF score < 8 points).

MNA(4)

The MNA scale is composed of 18 questions and rapid to measure items and can be performed in less than 15 minutes. The scale involves 4 parts: anthropometric assessment (BMI, arm and calf circumferences and weight loss), general assessment (6 questions related to lifestyle, medication and mobility), dietary assessment (8 questions related to number of meals, food and fluid intake and autonomy of feeding), and subjective assessment (self-perception of health and nutrition). The scoring categorizes subjects in the following manner: well-nourished (MNA score ≥ 24 points), at risk of malnutrition (MNA score = 17-23.5 points) and malnourished (MNA score < 17 points).

GNRI(5)

GNRI is a clinical biological index that is calculated as follows: 1.489 × serum albumin (g/L) + 41.7 × admission weight (kg)/ideal body weight (kg). The Lorentz formula was used to calculate ideal body weight according to the patients’ height and sex, as follows: [height (cm) − 100 − {(height (cm) − 150)/4}] for men and [height (cm) − 100 − {(height (cm) − 150)/2.5}] for women. GNRI is defined as 4 grades of nutrition-related risk: major risk (GNRI < 82), moderate risk (82 ≤ GNRI < 92), low risk (92 ≤ GNRI ≤ 98) and no risk (GNRI > 98).

**Supplementary Tables**

**Supplementary Table 1** The ability of nutrition-related indicators to distinguish AD from non-AD

|  | AUC | 95% CI | *P* | Youden Index | Cut-off | Sensitivity | Specificity | PPV | NPV |
| --- | --- | --- | --- | --- | --- | --- | --- | --- | --- |
| BMI | 0.63 | 0.57 - 0.69 | < 0.001** | 0.21 | 23.90 | 56.99 | 64.38 | 80.91 | 36.10 |
| Hip circumference | 0.62 | 0.54 - 0.69 | 0.007** | 0.25 | 95.00 | 41.67 | 83.33 | 81.83 | 44.23 |
| Calf circumference | 0.66 | 0.59 - 0.72 | < 0.001** | 0.35 | 35.00 | 58.55 | 76.12 | 84.76 | 44.74 |
| MNA | 0.72 | 0.66 - 0.77 | < 0.001** | 0.38 | 25.00 | 74.09 | 64.38 | 84.64 | 48.39 |
| GNRI | 0.72 | 0.66 - 0.78 | < 0.001** | 0.48 | 104.10 | 68.23 | 79.45 | 89.75 | 48.68 |
| Total protein | 0.80 | 0.74 - 0.84 | < 0.001** | 0.53 | 68.60 | 79.06 | 73.97 | 88.80 | 57.51 |
| Albumin | 0.80 | 0.74 - 0.84 | < 0.001** | 0.57 | 41.90 | 78.53 | 78.08 | 90.34 | 58.22 |
| Globulin | 0.68 | 0.62 - 0.73 | < 0.001** | 0.34 | 27.10 | 72.77 | 61.64 | 83.20 | 46.45 |
| Apolipoprotein A1 | 0.68 | 0.62 - 0.73 | < 0.001** | 0.33 | 1.65 | 83.68 | 49.32 | 81.09 | 53.78 |
| BMI + MNA + Total protein | 0.72 | 0.66 - 0.77 | < 0.001** | 0.41 | 0.65 | 78.01 | 63.01 | 84.63 | 52.33 |
| BMI + GNRI + Total protein | 0.72 | 0.66 - 0.76 | < 0.001** | 0.45 | 0.71 | 69.63 | 75.34 | 88.05 | 48.73 |
| Hip circumference + MNA + Total protein | 0.73 | 0.65 - 0.79 | < 0.001** | 0.38 | 0.57 | 72.90 | 65.00 | 78.81 | 57.33 |
| Hip circumference + GNRI + Total protein | 0.79 | 0.72 - 0.85 | < 0.001** | 0.60 | 0.67 | 68.22 | 91.67 | 93.60 | 61.77 |
| Calf circumference + MNA + Total protein | 0.75 | 0.69 - 0.81 | < 0.001** | 0.46 | 0.64 | 74.83 | 71.64 | 85.62 | 55.77 |
| Calf circumference + GNRI + Total protein | 0.76 | 0.70 - 0.82 | < 0.001** | 0.53 | 0.67 | 73.51 | 79.10 | 88.81 | 56.95 |
| BMI + MNA + Albumin | 0.81 | 0.75 - 0.85 | < 0.001** | 0.62 | 0.67 | 85.34 | 76.71 | 90.53 | 66.72 |
| BMI + GNRI + Albumin | 0.81 | 0.76 - 0.85 | < 0.001** | 0.55 | 0.73 | 73.82 | 80.82 | 90.95 | 54.19 |
| Hip circumference + MNA + Albumin | 0.83 | 0.76 - 0.88 | < 0.001** | 0.63 | 0.70 | 74.77 | 88.33 | 91.96 | 66.23 |
| Hip circumference + GNRI + Albumin | 0.81 | 0.74 - 0.86 | < 0.001** | 0.58 | 0.68 | 66.36 | 91.67 | 93.43 | 60.41 |
| Calf circumference + MNA + Albumin | 0.83 | 0.77 - 0.88 | < 0.001** | 0.63 | 0.64 | 83.44 | 79.10 | 90.01 | 67.91 |
| Calf circumference + GNRI + Albumin | 0.82 | 0.76 - 0.87 | < 0.001** | 0.60 | 0.69 | 76.82 | 83.58 | 91.35 | 61.50 |
| BMI + MNA + Globulin | 0.74 | 0.68 - 0.79 | < 0.001** | 0.39 | 0.67 | 78.53 | 60.27 | 83.76 | 51.82 |
| BMI + GNRI + Globulin | 0.74 | 0.68 - 0.79 | < 0.001** | 0.43 | 0.69 | 75.92 | 67.12 | 85.77 | 51.64 |
| Hip circumference + MNA + Globulin | 0.75 | 0.67 - 0.81 | < 0.001** | 0.42 | 0.56 | 82.24 | 60.00 | 78.59 | 65.42 |
| Hip circumference + GNRI + Globulin | 0.80 | 0.73 - 0.86 | < 0.001** | 0.54 | 0.65 | 71.03 | 83.33 | 88.38 | 61.70 |
| Calf circumference + MNA + Globulin | 0.76 | 0.70 - 0.82 | < 0.001** | 0.46 | 0.65 | 75.50 | 70.15 | 85.10 | 55.92 |
| Calf circumference + GNRI + Globulin | 0.76 | 0.70 - 0.82 | < 0.001** | 0.50 | 0.68 | 72.19 | 77.61 | 87.92 | 55.28 |
| BMI + MNA + Apolipoprotein A1 | 0.77 | 0.71 - 0.82 | < 0.001** | 0.43 | 0.71 | 72.11 | 71.23 | 86.68 | 49.58 |
| BMI + GNRI + Apolipoprotein A1 | 0.76 | 0.71 - 0.81 | < 0.001** | 0.46 | 0.74 | 67.89 | 78.08 | 88.94 | 48.35 |
| Hip circumference + MNA + Apolipoprotein A1 | 0.76 | 0.69 - 0.82 | < 0.001** | 0.39 | 0.68 | 59.43 | 80.00 | 84.03 | 52.70 |
| Hip circumference + GNRI + Apolipoprotein A1 | 0.82 | 0.75 - 0.87 | < 0.001** | 0.52 | 0.79 | 55.66 | 96.67 | 96.73 | 55.19 |
| Calf circumference + MNA + Apolipoprotein A1 | 0.77 | 0.71 - 0.83 | < 0.001** | 0.46 | 0.72 | 66.67 | 79.10 | 87.71 | 51.49 |
| Calf circumference + GNRI + Apolipoprotein A1 | 0.79 | 0.72 - 0.84 | < 0.001** | 0.51 | 0.72 | 70.00 | 80.60 | 88.97 | 54.57 |

Abbreviations: AD, Alzheimer’s disease; BMI, body mass index; MNA, the Mini Nutritional Assessment; GNRI, Geriatric Nutritional Risk Index; AUC, area under the curve; CI, confidence interval; PPV, positive predictive value; NPV, negative predictive value. ^**^*P* < 0.01.

**Supplementary Table 2** The ability of nutrition-related indicators to distinguish NC from AD-MCI

|  | AUC | 95% CI | *P* | Youden Index | Cut-off | Sensitivity | Specificity | PPV | NPV |
| --- | --- | --- | --- | --- | --- | --- | --- | --- | --- |
| Hip circumference | 0.56 | 0.47 - 0.66 | < 0.001** | 0.16 | 94.50 | 29.17 | 86.67 | 63.64 | 60.47 |
| GNRI | 0.66 | 0.57 - 0.73 | < 0.001** | 0.37 | 104.32 | 58.57 | 78.08 | 71.93 | 66.28 |
| Total protein | 0.73 | 0.65 - 0.80 | < 0.001** | 0.42 | 68.20 | 67.61 | 73.97 | 71.65 | 70.18 |
| Albumin | 0.75 | 0.67 - 0.82 | < 0.001** | 0.51 | 41.90 | 72.86 | 78.08 | 76.12 | 75.00 |
| Globulin | 0.65 | 0.56 - 0.73 | 0.001** | 0.32 | 27.10 | 70.00 | 61.64 | 63.63 | 68.18 |
| Apolipoprotein A1 | 0.65 | 0.56 - 0.73 | 0.001** | 0.31 | 1.65 | 81.43 | 49.32 | 60.64 | 73.47 |
| Hip circumference + GNRI + Total protein | 0.71 | 0.61 - 0.79 | < 0.001** | 0.42 | 0.50 | 53.19 | 88.33 | 78.10 | 70.69 |
| Hip circumference + GNRI + Albumin | 0.76 | 0.66 - 0.83 | < 0.001** | 0.51 | 0.54 | 59.57 | 91.67 | 84.86 | 74.32 |
| Hip circumference + GNRI + Globulin | 0.71 | 0.62 - 0.79 | < 0.001** | 0.39 | 0.48 | 55.32 | 83.33 | 72.22 | 70.42 |
| Hip circumference + GNRI + Apolipoprotein A1 | 0.73 | 0.64 - 0.81 | < 0.001** | 0.37 | 0.58 | 48.94 | 88.33 | 76.67 | 68.83 |

Abbreviations: NC, normal cognition; AD, Alzheimer’s disease; MCI, mild cognitive impairment; GNRI, Geriatric Nutritional Risk Index; AUC, area under the curve; CI, confidence interval; PPV, positive predictive value; NPV, negative predictive value. ^**^*P* < 0.01.

**Supplementary Table 3** The ability of nutrition-related indicators to distinguish AD-MCI from AD-D

|  | AUC | 95% CI | *P* | Youden Index | Cut-off | Sensitivity | Specificity | PPV | NPV |
| --- | --- | --- | --- | --- | --- | --- | --- | --- | --- |
| Calf circumference | 0.65 | 0.57 - 0.73 | < 0.001** | 0.25 | 33.50 | 51.06 | 74.14 | 76.16 | 48.36 |
| MNA-SF | 0.69 | 0.62 - 0.76 | < 0.001** | 0.31 | 13.00 | 93.39 | 37.50 | 71.52 | 77.14 |
| MNA | 0.72 | 0.65 - 0.78 | < 0.001** | 0.35 | 24.50 | 74.38 | 61.11 | 76.28 | 58.66 |
| Homocysteine | 0.65 | 0.58 - 0.72 | < 0.001** | 0.26 | 11.84 | 61.34 | 64.29 | 74.52 | 49.41 |
| Calf circumference + MNA-SF + Homocysteine | 0.74 | 0.66 - 0.80 | < 0.001** | 0.41 | 0.56 | 78.49 | 62.50 | 77.65 | 63.65 |
| Calf circumference + MNA + Homocysteine | 0.77 | 0.69 - 0.83 | < 0.001** | 0.43 | 0.67 | 62.37 | 80.36 | 84.05 | 56.27 |

Abbreviations: AD, Alzheimer’s disease; MCI, mild cognitive impairment; MAN-SF, the Mini Nutritional Assessment-short form; MNA, the Mini Nutritional Assessment; AUC, area under the curve; CI, confidence interval; PPV, positive predictive value; NPV, negative predictive value. ^**^*P* < 0.01.

**References**

1. Stratton RJ, Hackston A, Longmore D, Dixon R, Price S, Stroud M, et al. Malnutrition in Hospital Outpatients and Inpatients: Prevalence, Concurrent Validity and Ease of Use of the 'Malnutrition Universal Screening Tool' ('Must') for Adults. *The British journal of nutrition* (2004) 92(5):799-808. Epub 2004/11/10. doi: 10.1079/bjn20041258.

2. Kondrup J, Rasmussen HH, Hamberg O, Stanga Z. Nutritional Risk Screening (Nrs 2002): A New Method Based on an Analysis of Controlled Clinical Trials. *Clinical nutrition (Edinburgh, Scotland)* (2003) 22(3):321-36. Epub 2003/05/27. doi: 10.1016/s0261-5614(02)00214-5.

3. Kaiser MJ, Bauer JM, Ramsch C, Uter W, Guigoz Y, Cederholm T, et al. Validation of the Mini Nutritional Assessment Short-Form (Mna-Sf): A Practical Tool for Identification of Nutritional Status. *The journal of nutrition, health & aging* (2009) 13(9):782-8. Epub 2009/10/09. doi: 10.1007/s12603-009-0214-7.

4. Guigoz Y, Vellas B, Garry PJ. Assessing the Nutritional Status of the Elderly: The Mini Nutritional Assessment as Part of the Geriatric Evaluation. *Nutrition reviews* (1996) 54(1 Pt 2):S59-65. Epub 1996/01/01. doi: 10.1111/j.1753-4887.1996.tb03793.x.

5. Bouillanne O, Morineau G, Dupont C, Coulombel I, Vincent JP, Nicolis I, et al. Geriatric Nutritional Risk Index: A New Index for Evaluating at-Risk Elderly Medical Patients. *The American journal of clinical nutrition* (2005) 82(4):777-83. Epub 2005/10/08. doi: 10.1093/ajcn/82.4.777.
